# Thulium laser enucleation of prostate versus laparoscopic trans-vesical simple prostatectomy in the treatment of large benign prostatic hyperplasia: head-to-head comparison

**DOI:** 10.1590/S1677-5538.IBJU.2021.0726

**Published:** 2022-01-11

**Authors:** Riccardo Bertolo, Orietta Dalpiaz, Giorgio Bozzini, Chiara Cipriani, Matteo Vittori, Thomas Alber, Francesco Maiorino, Marco Carilli, Robin Zeder, Valerio Iacovelli, Michele Antonucci, Marco Sandri, Pierluigi Bove

**Affiliations:** 1 San Carlo di Nancy Hospital Department of Urology Rome Italy Department of Urology, San Carlo di Nancy Hospital, Rome, Italy; 2 LKH Hochsteiermark Department of Urology Leoben Austria Department of Urology, LKH Hochsteiermark, Leoben, Austria; 3 ASST Valle Olona Department of Urology Busto Arsizio Varese Italy Department of Urology, ASST Valle Olona, Busto Arsizio, Varese, Italy; 4 Tor Vergata University of Rome Urology Unit Department of Surgery Rome Italy Urology Unit, Department of Surgery, Tor Vergata University of Rome, Rome, Italy; 5 University of Brescia Big & Open Data Innovation Laboratory Italy Big & Open Data Innovation Laboratory (BODaI-Lab), University of Brescia, Italy

**Keywords:** Prostatectomy, Thulium, Prostatic Hyperplasia

## Abstract

**Objectives:**

To compare thulium laser enucleation of prostate (ThuLEP) versus laparoscopic trans-vesical simple prostatectomy (LSP) in the treatment of benign prostatic hyperplasia (BPH).

**Materials and Methods:**

Data of patients who underwent surgery for “large” BPH (>80mL) at three Institutions were collected and analyzed. Two institutions performed ThuLEP only; the third institution performed LSP only. Preoperative (indwelling catheter status, prostate volume (PVol), hemoglobin (Hb), Q_max_, post-voiding residual volume (PVR), IPSS, QoL, IIEF-5) and perioperative data (operative time, enucleated adenoma, catheterization time, length of stay, Hb-drop, complications) were compared. Functional (Q_max_, PVR, %ΔQ_max_) and patient-reported outcomes (IPSS, QoL, IIEF-5, %ΔIPSS, %ΔQoL) were compared at last follow-up.

**Results:**

80 and 115 patients underwent LSP and ThuLEP, respectively. At baseline, median PVol was 130 versus 120mL, p <0.001; Q_max_ 9.6 vs. 7.1mL/s, p=0.005; IPSS 21 versus 25, p <0.001. Groups were comparable in terms of intraoperative complications (1 during LSP vs. 3 during ThuLEP) and transfusions (1 per group). Differences in terms of operative time (156 vs. 92 minutes, p <0.001), Hb-drop (-2.5 vs. −0.9g/dL, p <0.001), catheterization time (5 vs. 2 days, p <0.001) and postoperative complications (13.8% vs. 0, p <0.001) favored ThuLEP. At median follow-up of 40 months after LSP versus 30 after ThuLEP (p <0.001), Q_max_ improved by 226% vs. 205% (p=0.5), IPSS decreased by 88% versus 85% (p=0.9), QoL decreased by 80% with IIEF-5 remaining almost unmodified for both the approaches.

**Conclusions:**

Our analysis showed that LSP and ThuLEP are comparable in relieving from BPO and improving the patient-reported outcomes. Invasiveness of LSP is more significant.

## INTRODUCTION

Lower urinary tract symptoms (LUTS) secondary to benign prostatic hyperplasia (BPH) are among the most common medical issues for the aging male (1). Indeed, population-based studies have suggested that more than 40% of men aged over 60 years old suffers from BPH (2).

In patients bothered by LUTS secondary to benign prostatic obstruction (BPO) refractory to medical therapy and indication to surgical treatment, transurethral resection of the prostate (TURP) and open simple prostatectomy have been the historical reference-standards for prostates <80g and ≥80 grams, respectively (3).

Both TURP and open simple prostatectomy have demonstrated effectiveness in relieving from the BPO and able to offer durable improvements in urinary functional outcomes (4, 5).

However, they suffer from the drawback of potential perioperative morbidity. Looking for a reduction of such surgical morbidity, a variety of minimally-invasive alternative techniques have been introduced.

In this evolving scenario, the thulium laser enucleation of prostate (ThuLEP) has been described in 2010 by Herrmann et al. who pioneered the concept of an anatomical enucleation of the prostate adenoma by a widely blunt dissection of the transitional zone of the gland (6). Nowadays, ThuLEP represents a viable option in case of large (>80mL) BPH as suggested by the European Association of Urology (EAU) guidelines (7).

Experienced laparoscopic surgeons are still challenging endoscopic enucleation techniques with laparoscopic simple prostatectomy (LSP), performed via a trans-vesical or a trans-capsular approach, either pure or robot-assisted, which duplicates the principles of open adenomectomy (8, 9).

While several analyses have been published comparing LSP to the holmium laser enucleation (HoLEP) and to the green-laser enucleation of prostate, to the best of our knowledge, no study has been focused on the comparison of ThuLEP versus LSP. To contribute to this field, the present study was conceived aimed to compare the perioperative outcomes of ThuLEP versus LSP in the treatment of large BPH.

## MATERIALS AND METHODS

Data of consecutive patients who underwent surgery for large BPH (configured as prostate volume >80mL at preoperative imaging) at three referral Institutions were collected on dedicated prospectively maintained databases and retrospectively analyzed. The study was approved by the leading institution local ethics committee (no. approval STS CE Lazio 1/N-726) and performed in accordance with the ethical standards as laid down in the 1964 Declaration of Helsinki and its later amendments.

Two institutions performed ThuLEP only (ThuLEP Group) between September 2018 and April 2019; the third institution performed LSP only (LSP Group) via a trans-vesical approach. LSP procedures were performed between October 2012 and September 2019. All the procedures were performed by experienced surgeons. Surgical techniques for LSP and ThuLEP have been previously described (10, 11). For LSP, an extra-peritoneal approach was chosen. After dissecting the prostate and the bladder free from the overlying fatty tissue, a 3-4cm vertical cystotomy incision was performed at the level of the bladder neck. The ureteral orifices were identified, then the enucleation the adenoma was carried out within the plane between the prostatic capsule and the adenoma. Bladder neck trigonization was performed to facilitate re-epithelialization. A three-ways 22F Dufour catheter was introduced and inflated, and cystotomy was closed using absorbable barbed sutures in double layer.

ThuLEP was performed according to either a two-lobes or a three-lobes technique, depending on the anatomy of the adenoma. The procedure was performed using an Iglesias 26F resectoscope, with a 4mm, 12 degrees optics. The 200 Watt-Cyber-TM laser generator (Quanta System) was used with maximum power of 70 Watts set for cutting and 40 Watts set for coagulation. Low-power coagulation was used during almost the whole enucleation, while activating cutting in case of sticky tissue. A 550nm laser fiber was used, with apical release performed at the beginning. The enucleated lobes were morcellated by the Piranha morcellator (Richard Wolf).

### Outcome measurements

Preoperative variables, including age, body mass index (BMI), comorbidities as assessed by the Charlson's index (CCI) (12), prostate volume (PVol as assessed by trans-rectal ultrasonography - TRUS), hemoglobin (Hb), serum prostate specific antigen (PSA), maximum urinary flow rate at uroflowmetry (Q_max_) with post-voiding residual volume (PVR) (or alternatively an indwelling catheter status), International Prostate Symptom Score (IPSS) with Quality of Life (QoL) and International Index of Erectile Function questionnaire (IIEF-5) were compared at baseline (13). In case of elevated serum PSA and suspected prostate cancer, TRUS biopsy was performed to exclude malignancy, as appropriate.

Perioperative data, including intraoperative complications, operative time, enucleated adenoma grams at final pathology, catheterization time, length of stay, PSA at three months postoperatively, Hb drop (either absolute and percentage), and complications classified according to the Clavien-Dindo grade (14), were compared.

Functional data, including Q_max_, PVR, and %ΔQ_max_, together with patient-reported outcomes as assessed by the IPSS with QoL questionnaire (with relative %ΔIPSS and %ΔQoL), were compared at the last follow-up available. Finally, IIEF-5 questionnaire was re-assessed at the last follow-up available to check for potential impact of the treatment received on sexual function.

### Statistical Analysis

Continuous variables were summarized using medians and interquartile ranges (IQR); frequencies and proportions were used to summarize categorical variables. Median values of continuous variables calculated in the treatment groups were compared by using the two-sample Wilcoxon rank-sum test, while differences in frequencies and proportions of categorical variables were compared by using the Fisher's exact test.

For continuous intraoperative outcomes, the mean difference between groups, after adjusting for potential confounders (including age, BMI, Hb, PSA, PVol, IPSS, QoL, IIEF-5 and treatment received), was estimated by using multivariable linear regression models. Postoperative complications were compared between the groups, after adjusting for potential confounders, by using multivariable exact logistic regression models.

Significance level was set at p-value <0.05. Statistical analysis was performed by using Stata 16.1 (StataCorp. 2019. Stata Statistical Software: Release 15. College Station, TX: StataCorp LLC).

## RESULTS

Eighty versus 115 patients underwent LSP versus ThuLEP and were analyzed. Groups were comparable at baseline in age, Charlson's comorbidity index and PVR. Statistically significant differences were found in BMI, percentage of patients with indwelling catheter, PSA, Hb, PVol (130 (IQR 115-150) versus 120 (100-160mL), LSP versus ThuLEP, respectively, p <0.001), _Qmax_ (9.6 (IQR 5.9-11.3) vs. 7.1 (5.9-8.7mL/s), p=0.005), IPSS (21 (IQR 16-27) vs. 25 (23-27), p <0.001), QoL, and IIEF-5 at univariate analysis. Table-1 reports the complete data about the baseline characteristics of patients stratified by treatment group.

**Table 1:** Distribution of baseline characteristics of patients in the treatment groups.

		Group LSP n = 80	Group ThuLEP n = 115	p-value
Age, years		69 (65 – 74)	68 (64 – 73)	0.5
Charlson's Comorbidity Index				0.7
	0-1	80 (100)	112 (97.4)	
	≥ 2	0 (0)	3 (2.6)	
BMI		27.0 (24.4 – 28.7)	25.0 (23.3 – 27.0)	< 0.001
Indwelling catheter		35 (43.8)	16 (13.9)	< 0.001
PSA, ng/mL		11.0 (4.9 – 15.8)	3.1 (2.3 – 5.6)	< 0.001
Hemoglobin, g/dL		14.9 (14.2 – 15.8)	14.2 (13.4 – 15.6)	0.003
Prostate Volume, mL		130 (115 – 150)	120 (100 – 160)	< 0.001
Qmax, mL/s		9.6 (5.9 – 11.3)	7.1 (5.9 – 8.7)	0.005
PVR, mL		100 (50 – 200)	90 (75 – 120)	0.09
IPSS		21 (16 – 27)	25 (23 – 27)	< 0.001
QoL		5 (4 – 5)	6 (5 – 6)	< 0.001
IIEF-5		15 (6 – 22)	19 (16 – 23)	< 0.001

Median is reported for continuous variables, while number of observations is reported for categorical variables. Inter-Quartile Range (IQR) and percentages are reported in brackets, as appropriate. LSP: Laparoscopic Simple Prostatectomy; ThuLEP: Thulium Laser Enucleation of Prostate; BMI: Body Mass Index; PSA: Prostate Specific Antigen; Qmax: Maximum Urinary Flow; PVR: Post-Voiding Residual volume; IPSS: International Prostate Symptom score; QoL: Quality of Life; IIEF-5: International Index of Erectile Function Questionnaire.

Concerning the perioperative data (Table-2), the treatment groups were comparable in terms of intraoperative complications (1 vs. 3, namely 1 conversion to open surgery during LSP vs. 2 extravasation syndromes and 1 severe bleeding occurred during ThuLEP) and transfusions (1 patient per group). Resected adenoma grams were comparable between the techniques (83, IQR 70-104 vs. 85, IQR 67-118, p=0.7).

**Table 2:** Distribution of peri-operative and post-operative outcomes in the treatment groups.

		Group LSP n = 80	Group ThuLEP n = 115	p-value
Operative time, min		156 (134 – 193)	92 (70 – 110)	< 0.001
Intraoperative Complications		1 (1.3)	3 (2.6)	0.6
Resected Adenoma, grams		83 (70 – 104)	85 (67 – 118)	0.7
Hemoglobin at 1^st^ postoperative day, g/dL		12.5 (11.5 – 13.2)	13.1 (12.5 – 14.2)	< 0.001
Δ Hemoglobin at 1^st^ postoperative day, g/dL		-2.5 (-3.0 – −1.8)	-0.9 (-1.7 – −0.3)	< 0.001
% Δ Hemoglobin at 1^st^ postoperative day		-16.6 (-21.1 – −12.3)	-6.0 (-11.5 – −2.3)	< 0.001
Postoperative Complications*		11 (13.8)	0 (0)	< 0.001
	1	6 (7.5)	-	
	2	3 (3.7)	-	
	3b	1 (1.3)	-	
	4a	1 (1.3)	-	
Transfusions		1 (1.3)	1 (0.9)	0.8
Catheterization Time, days		5 (5-7)	2 (1-3)	< 0.001
Hospital Stay, days		9 (8-9)	2 (2-3)	< 0.001
PSA at 3^rd^ Postoperative Month, ng/mL		1.0 (0.4 – 2.1)	1.8 (1.2 – 2.5)	< 0.001
Δ PSA at 3^rd^ Postoperative Month, ng/mL		-9.1 (-14.0 – −4.0)	-1.4 (-3.5 – −0.6)	< 0.001
% Δ PSA at 3^rd^ Postoperative Month		-90 (-96 – −78)	-46 (-70 – −26)	< 0.001
Q_max_, mL/s		30.2 (22.2 – 39.8)	22.2 (19.5 – 26.4)	< 0.001
Δ Q_max_, mL/s		21.2 (14.2 – 30.8)	15.5 (12.0 – 18.9)	0.037
% Δ Qmax		226 (172 – 335)	205 (152 – 295)	0.5
PVR, mL		0 (0 – 10)	0 (0 – 30)	0.004
IPSS		3 (1 – 6)	4 (2 – 6)	0.1
Δ IPSS		-16 (-25 – −11)	-21 (-24 – −17)	0.04
% Δ IPSS		-88 (-93 – −63)	-85 (-92 – −77)	0.9
QoL		1 (0 –1)	1 (1 – 2)	0.037
Δ QoL		-3 (-5 – −2.5)	-4 (-5 – −3)	0.003
% Δ QoL		-80 (-100 – −67)	-80 (-92 – −67)	1
IIEF-5		11 (5 – 21)	18 (14 – 21)	< 0.001
Δ IIEF-5		0 (0 – 0)	0 (-4 – 1)	0.5
% Δ IIEF-5		0 (0 – 0)	0 (-23.8 – 11.1)	0.3

* According to Clavien-Dindo Classification.

Inter-Quartile Range (IQR) and percentages were reported in brackets, as appropriate. Qmax: Maximum Urinary Flow; PVR: Post-Voiding Residual volume; IPSS: International Prostate Symptom score; QoL: Quality of Life; EPIC: Expanded Prostate Cancer Index Composite

Median is reported for continuous variables, while number of observations is reported for categorical variables. Inter-Quartile Range (IQR) and percentages are reported in brackets, as appropriate.

LSP = Laparoscopic Simple Prostatectomy; ThuLEP = Thulium Laser Enucleation of Prostate; PSA = Prostate Specific Antigen; Qmax = Maximum Urinary Flow; PVR = Post-Voiding Residual volume; IPSS = International Prostate Symptom score; QoL = Quality of Life; IIEF-5 = International Index of Erectile Function Questionnaire.

Significant differences were found in terms of operative time (156 vs. 92 minutes, LSP vs. ThuLEP, respectively, p <0.001), Hb drop at the 1st postoperative day (-2.5 vs. −0.9g/dL, p <0.001), catheterization time (5 vs. 2 days, p <0.001), hospital stay (9 vs. 2 days, p <0.001), and postoperative complications (13.8% vs. 0, p <0.001). Post-operative complications recorded after LSP included 2 arrhythmias, 2 epididymitis, 4 urine leakages, 2 peri-vesical hematomas and 1 severe acute bleeding, the latter being managed with trans-urethral hemostasis (Clavien 3b).

At the third month postoperatively, PSA was 1.0 (IQR 0.4-2.1) vs. 1.8 (1.2-2.5ng/mL) (LSP vs. ThuLEP, respectively, p <0.001), with a more pronounced decrease after LSP (-90% vs. −46%, p <0.001).

At a median follow-up of 40 months (IQR 25-56) after LSP vs. 30 months (30-30) after ThuLEP (p <0.001), no significant differences were found between the approaches in terms of improvement in functional outcomes and patient-reported outcomes. Namely, Q_max_ was found improved by 226% (IQR 172-335) vs. 205% (152-295) (p=0.5), reaching a median value of 30.2mL/s (IQR 22.2-39.8) vs. 22.2mL/s (19.5-26.4) (p <0.001); PVR became virtually absent in both the groups; IPSS decreased by 88% (IQR 93-63) vs. 85% (92-77) (p=0.9), reaching a median value of 3 (IQR 1-6) vs. 4 (2–6) (p=0.1); QoL decreased by 80% (IQR 100-67) vs. 80% (92-67) (p=1), reaching a value of 1 (0-1) vs. 1 (1–2) (p=0.037). IIEF-5 remained almost unmodified regardless of the approach (0 (IQR 0-0) vs. 0 (-4; 1), LSP versus ThuLEP, respectively, p=0.5).

After multivariable analyses, the adjusted average difference of Hb drop between the treatment groups (LSP vs. ThuLEP) was 1.1g/dL (95% C.I. 0.7-1.5, p <0.001). This value indicates that the reduction of Hb after surgery was significantly lower in the ThuLEP group compared to that observed in the LSP group (-1.3g/dL vs. −2.4g/dL, respectively).

The adjusted average catheterization time in the LSP and the ThuLEP groups were 5.8 and 2.7 days, respectively; the adjusted mean difference between the groups was −3.1 days (-3.9 to −2.3 days, p <0.001). The number of complications observed in the LSP group was 11 (13.8%) and 0 for ThuLEP.

The difference between the two rates was statistically significant even after adjusting for potential confounders (p <0.006).

## DISCUSSION

Data from the present comparative analysis between LSP and ThuLEP showed that the techniques are comparable in relieving from BPO and improving the patient-reported outcomes. On the other hand, our analysis found that ThuLEP is characterized by a lower invasiveness, given that patients who underwent LSP were more likely to experience a more consistent postoperative Hb drop, were more likely to report complications and were more likely to keep the catheter for a longer time.

Anecdotal literature compared simple prostatectomy, whatever the surgical approach, to the adoption of thulium laser fibers during endourological procedures to treat BPO. One multicentric three-match comparative analysis was published by Nestler et al. (15). In this study, the data of 35 robot-assisted LSP patients, of 390 open simple prostatectomy patients and of 937 thulium vapo-enucleation patients were collected at referral institutions and matched by age, PVol, Qmax, IPSS and QoL. As a result, compared to open and robotic LSP, thulium vapo-enucleation showed a shorter operative time, with robotic LSP showing longer operative time (83 vs. 130 vs. 182 min, p <0.004). Open surgery suffered from higher blood losses, transfusion's and complication's rates. Such data are in line to what observed in our multicenter experience. Nestler et al. did not find any significant difference in terms of Hb drop (1.2 vs. 1.5g/dL, p=0.2) and related transfusions rate (0 vs. 9.4%, p=0.4) between endoscopic and robotic approach. In both the groups, one patient underwent re-intervention due to acute bleeding. In our analysis, absolute and percentage Hb drop were higher after LSP, even after adjusting for potential confounders. As regarding the postoperative Q_max_ and the patients reported outcomes, no differences were found among the three techniques investigated in the Nestler and colleague's analysis, that is again in line with the herein reported findings.

Another study in this setting was published by Enikeev et al., who concluded that ThuLEP does represent a minimally-invasive treatment modality for the management of BPO in large volume glands (>80cc) (16). The authors compared the data of 40 patients who underwent open simple prostatectomy to those of 90 patients who underwent ThuLEP. With treatment groups being comparable at baseline, the authors found no significant differences as regarding the operative time, and the weight of adenomatous tissue removed. On the other hand, patients who underwent open simple prostatectomy had significantly longer catheterization time and hospital stay (9.0 vs. 3.3 days, p <0.001). Such data are comparable to our experience: our data overlaps those published by Enikeev et al., if excluding the operative time that we found longer for LSP due to the intrinsic challenges related to the pure laparoscopic approach when duplicating the open adenomectomy technique.

Similar experiences were published but investigating a matured alternative to ThuLEP with more consistent literature. Gunseren et al. compared the data of 60 HoLEP versus 61 LSP versus 37 open simple prostatectomy patients. While HoLEP was found comparable to open prostatectomy (89.6±27.4 vs. 95.9±25.1 min), LSP had a statistically longer operative time (124.8±40.2, p <0.001), that was not dissimilar to the one observed in our LSP group (17). No significant differences were found in terms of peri-operative complication's and transfusion's rates. The improvements in Q_max_ (19.6±6 vs. 18.6±5.2 vs. 16.7±4.8mL/s, p=0.052, respectively) and IPSS (18.3±5 vs. 16.7±4.8 vs. 16.3±4, p=0.07, respectively) at the 3rd month postoperatively were comparable among the groups, again confirming that, when properly performed, “enucleation is enucleation” (18). Similar outcomes were obtained by Juaneda et al., who compared the data of 20 HoLEP vs. 20 LSP patients reporting comparable short-term functional results and complication rates between the approaches, with the advantage of shorter catheterization time and hospital stay favoring HoLEP (with a relatively higher cost-effectiveness) (19).

In summary, after reviewing similar studies about the topic, if compared to the virtually “ideal” reference standard of simple prostatectomy, the endoscopic transurethral laser enucleation of a large prostate adenoma (performed either by using the holmium or the thulium lasers) appears to be equally effective in de-obstructing the patient, as witnessed by the significant improvement in the uroflowmetry parameters and the patient-reported outcomes. Moreover, our analysis shows that the invasiveness related to the treatment ThuLEP itself is reduced, as the reader can deduct from the lower drop in Hb, the shorter catheterization time, and the lower rate of complications.

As with all the studies, there are several limitations to our analysis. First, there is the potential for selection bias that remained not accounted. Given the exclusiveness of each institution for its specific technique, patients could have self-selected to the surgical or endoscopic indication.

Second, a standardized post-operative management was not utilized which likely caused some heterogeneity between the groups regardless of the surgical treatment received. On the other hand, each of the involved surgeons performed a technique he/she was confident with and offered the patients the best performance in his/her hands.

Third, several differences at baseline were found by univariate statistical analysis. Although most of them were considered of limited clinical relevance, the reader should bear in mind their existence and their potential impact on the outcomes. Moreover, the key data for assessing the success of the de-obstructive techniques herein investigated were compared at different time points, due to differences in the available follow-up between the treatment groups. However, if “enucleation is enucleation”, as witnessed by the comparable resected adenoma grams whatever the adopted technique, we would expect similar results even after ThuLEP matured longer follow-up.

Fourth, we recognize the relatively small populations analysed in our research, but the specific indication to minimally-invasive surgery for large BPH, particularly to pure LSP is still a niche in urology, worth of being investigated. Similar studies about the topic have been published, but none specifically compared LSP versus ThuLEP in a head-to-head fashion.

Lastly, the “elephant in the room” is the reporting of urinary incontinence. This is arguably one of the reasons why transurethral enucleation procedures have failed to gain as much traction and diffusion as should have been expected considering the number of years this approach was described and the perioperative outcomes. Moreover, the occurrence of bladder neck contracture and/or urethral stenosis is worth of being investigated. We are performing a dedicated analysis focused on the incidence and the risk factors for bladder neck contracture and/or urethral stenosis after ThuLEP.

## CONCLUSION

Data from the present comparative analysis showed that LSP and ThuLEP are comparable in relieving from BPO and improving the patient-reported outcomes. ThuLEP resulted as a less invasive approach, given that patients who underwent LSP were more likely to experience more consistent postoperative Hb drop, to keep the catheter longer, and to experience complications..
